# Process Evaluation of a Personality Targeted Intervention for Addictive Eating in Australian Adults

**DOI:** 10.3390/bs10120186

**Published:** 2020-12-03

**Authors:** Yive Yang, Li Kheng Chai, Rebecca Collins, Mark Leary, Megan Whatnall, Tracy Burrows

**Affiliations:** 1 School of Health Sciences, Faculty of Health and Medicine, University of Newcastle, Callaghan, NSW 2308, Australia; yive.yang@uon.edu.au (Y.Y.); rebecca.collins10@uon.edu.au (R.C.); mark.leary@uon.edu.au (M.L.); megan.whatnall@newcastle.edu.au (M.W.); 2 Priority Research Centre for Physical Activity and Nutrition, University of Newcastle, Callaghan, NSW 2308, Australia; 3 Health and Wellbeing Queensland, Queensland Government, Milton, QLD 4064, Australia; likheng.chai@qut.edu.au; 4 Centre for Children’s Health Research, Institute of Health and Biomedical Innovation (IHBI) Exercise and Nutrition, Queensland University of Technology, South Brisbane, QLD 4101, Australia

**Keywords:** feasibility, addictive eating, food addiction, behaviour

## Abstract

Addictive eating prevalence is estimated at 15–20% in studied populations, and is associated with concurrent mental health conditions and eating disorders as well as overweight and obesity. However, few evidence-based interventions targeting addictive eating are available. The further development of evidence-based interventions requires assessment of intervention feasibility and efficacy. This study aimed to determine the feasibility, including intervention delivery and program acceptability, of FoodFix; a personality targeted intervention for the treatment of addictive eating behaviours in Australian adults. Participants (n = 52) were randomised to intervention (n = 26) or wait-list control groups (n = 26) and received three personalised telehealth sessions with an Accredited Practising Dietitian over seven weeks. Intervention delivery was assessed by tracking adherence to scheduled timing of intervention sessions. Program acceptability of participants was assessed via an online process evaluation survey and program acceptability of intervention providers was assessed via semi-structured phone interviews. In total, 79% of participants adhered to scheduled timing for session two and 43% for session three, defined as within one week (before/after) of the scheduled date. Further, 21% of participants completed the process evaluation survey (n = 11). The majority of participants were extremely/very satisfied with FoodFix (n = 7, 63%). Intervention providers (n = 2) expressed that they felt adequately trained to deliver the intervention, and that the overall session format, timing, and content of FoodFix was appropriate for participants. These findings highlight the importance of assessing intervention feasibility to further understand intervention efficacy.

## 1. Introduction

Increasing evidence posits that certain individuals are susceptible to addictive patterns of eating or addictive eating, particularly associated with highly palatable, processed, and energy-dense foods [1,2,3,4]. However, addictive eating is not currently a recognized medical disorder under the Diagnostic and Statistical Manual of Mental Disorders (DSM-5) [5]. Instead, addictive eating has been commonly measured and assessed through the use of self-report surveys such as the Yale Food Addiction Scale (YFAS) [4,5,6,7]. Systematic reviews have suggested that the mean prevalence of addictive eating, as identified using the YFAS, is approximately 15–20% in studied populations around the world, and approximately 11% in Australian adults [8], but this varies greatly between different groups [9,10]. The prevalence of food addiction is higher for example among females than males, in individuals with overweight/obesity compared with healthy weight individuals, and in individuals with a diagnosed eating disorder than those without [9,10]. Addictive eating has also been reported to be much higher in individuals with concurrent mental health conditions such as depression, anxiety, and eating disorders, particularly binge eating disorder (BED) [9,10]. A higher YFAS symptom score, in addition to clinical impairment, is also associated with higher Body Mass Index (BMI) as well as increased body and trunk fat percentage [9,10,11].

Despite the high prevalence of addictive eating in the general population, and the severity of the associated conditions, there are limited treatment options available that are delivered by qualified health professionals, and instead, self-help groups are common [12]. A recent systematic review by Cassin et al. (2020) exploring psychosocial interventions for addictive eating included only eight studies, with only two of these studies specifically targeted to addictive eating [13]. The interventions in these two studies were abstinence-based, however the effect of these interventions on addictive eating symptoms is unclear due to the methodological limitations of the studies (i.e., did not include the same participants or measures over time). The remaining studies included an outcome measure of addictive eating but were targeted to the treatment of bulimia nervosa or overweight and obesity. Further, whilst a 2018 web search review of online support options for addictive eating identified 13 support groups, only three of these involved input from qualified health professionals [12]. These online support groups primarily involved 12-step programs based on similar approaches used in drug and alcohol addiction, had a focus on spirituality, and included food plans or lists of foods to abstain from eating. Overall, there is a need for evidence-based treatment options for addictive eating, especially given the expertise available in dietary approaches as well as addiction. Treatments are needed which integrate the best available evidence from research with clinical expertise and consideration of patient needs and preferences [14]. In terms of which evidence to apply to addictive eating treatment, a range of approaches could be investigated including behavioural or substance related treatment approaches, such as motivational interviewing [15,16]. There is also evidence demonstrating the links between personality traits and addictive eating, and therefore intervention approaches targeting personality traits (e.g., through providing coping strategies) may also be effective in addictive eating treatment [17].

Critical to the development of evidence-based addictive eating interventions is the assessment of intervention feasibility, including fidelity and acceptability [18,19]. Assessment of intervention fidelity is critical in determining internal and external validity, that is, whether the results are due to the intervention, and the feasibility of the intervention in a real life setting [20]. Assessment of the acceptability of an intervention to participants and providers is also integral to understanding intervention efficacy and to inform the refinement of future interventions [20].

The aim of this study was to determine the feasibility of FoodFix, the first personality targeted intervention for the treatment of addictive eating behaviours in Australian adults, including intervention delivery and program acceptability.

## 2. Methods

### 2.1. Study Design

A randomised controlled trial was conducted to assess the feasibility and preliminary efficacy of FoodFix when compared to a waitlist control group. The preliminary efficacy results for addictive eating and dietary intake have been previously published [21]. To assess feasibility, intervention delivery and program acceptability were assessed. Intervention delivery was assessed by tracking adherence to scheduled timing of intervention sessions. Participant perceptions of program acceptability were assessed via an online process evaluation survey and program acceptability to intervention providers were assessed via semi-structured phone interviews. Both were administered after completion of 3-month follow up measures. The process evaluation survey was modelled on that used in a previous study [22]. The trial was registered with the Australian New Zealand Clinical Trial Registry (ACTRN12619001540101) and received ethics approval from the University of Newcastle Human Research Ethics Committee (HREC 2017-0167).

### 2.2. Participants

Participants were recruited from February to July 2018. Recruitment was via media releases through social media (e.g., Twitter), newspaper and radio, and emailing a cohort from a previous research study about addictive eating who had agreed to be recontacted [23]. The timing of these multiple methods of recruitment was overlapping throughout the recruitment period in an attempt to boost the visibility of advertisements and therefore interest in participating. All recruitment materials were approved during the ethics approval process. Eligibility criteria were: individuals with addictive eating as determined using the modified YFAS (mYFAS), [24], score of <46 on the Binge Eating Scale (BES [25]), BMI > 25 kg/m^2^, and having access to the internet via desktop or mobile device. Exclusion criteria were: living outside of Australia, pregnant, and non-English speaking. Participants were limited to Australia as this was a small feasibility study, accommodating different time zones would have impacted on intervention delivery, and the food frequency questionnaire used to assess dietary intake and provide dietary feedback within the intervention was specific to Australian foods. Interested individuals completed a screening questionnaire to determine whether eligibility criteria were met, and eligible participants then completed baseline surveys via an online platform (Qualtrics). The screening questionnaire included the mYFAS and the baseline survey included the YFAS 2.0. Participants were then randomly allocated to the intervention group or a 3-month wait-list control group. Randomisation was generated by one researcher using block sequences produced using a computer random number generator. The allocation was concealed in an opaque envelope and given to the participant by another member of the research team by email/phone. The wait-list control group received the intervention after the 3-month follow-up.

### 2.3. Intervention

FoodFix aims to assist individuals with addictive eating to reduce their overall frequency of overeating episodes and to improve their dietary intake and behaviours. The intervention included three telehealth sessions delivered over 3 months. The sessions were delivered by Accredited Practicing Dietitians (APD) with extensive clinical experience through the online platform VSee or over the phone if VSee was not accessible. Participants were also emailed a session summary following each session. Sessions were booked in by participants using an online scheduling appointment system (Acuity). Session timing and duration were; session one (week one—baseline, 45 min), session two (week three, 25–30 min), and session three (week seven, 15–20 min). A description of the intervention sessions has been previously published [21] and is available as (Supplementary Material (Table S1). Broadly, session one focused on exploring each participant’s reasons for participating in the study, their experience of addictive eating and goal setting focused around dietary intake, session two focused on personality traits and coping strategies, and session three was a check-in focusing on problem solving any barriers and providing encouragement relating to goals and coping strategies. FoodFix was personalised by tailoring the content to participants dominant personality traits, assessed at baseline using the Substance Use Risk Profile Scale [26] (e.g., providing specific coping strategies based on personality type), and those delivering the intervention incorporated motivational interviewing to assist in achieving goals. FoodFix was developed as an adaptation of an existing intervention for alcohol addiction, the Quik Fix Personality-targeted Intervention [17], and was based on social cognitive theory (SCT) [27]. FoodFix was designed as a brief intervention (i.e., ≤4 sessions) substantiated by the efficacy of this approach in treating substance addiction [28].

### 2.4. Training of Intervention Providers

A standardised FoodFix Manual was created which contained a guide for the structure and content of the telehealth consultation sessions. The manual was developed by experienced APD’s and psychologists (T.B., M.R., R.C., L.H.) through adapting the Quik Fix Personality-targeted Intervention Treatment Manual [17], and incorporating brief motivational interviewing and personality-targeted intervention strategies that are often used in other forms of addiction counselling [17]. One APD (R.C.), experienced and trained in motivational interviewing, was initially responsible for delivering the telehealth sessions. A second APD (M.L.), also experienced in motivational interviewing, was then trained to deliver the FoodFix program through: (1) one face-to-face meeting to discuss the overall project where M.L. was provided with the FoodFix Manual to review; (2) a second face-to-face meeting to answer any questions and to discuss the delivery of the intervention, and; (3) sitting in on two baseline (week one) sessions and two follow up (week three) sessions run by R.C. M.L. then conducted a baseline session independently and debriefed with R.C. regarding session content delivery via phone and email.

### 2.5. Measures

#### 2.5.1. Intervention Delivery

Intervention delivery was assessed by comparing the scheduled session dates outlined in the protocol with that recorded in the online scheduling appointment system (Acuity). Adherence to intervention delivery scheduling was defined as sessions that were conducted within one week (before or after) of the scheduled session date. Adherence to scheduled timing is only relevant for sessions two and three. There was no scheduled timing for session one because of the variability in time taken to complete screening and baseline surveys. The timing of session one was at the earliest convenience for participant and intervention provider following randomisation. Additionally, for wait-list control group participants who completed the intervention after the 3-month follow up, the timing of session one was relative to having completed the 3-month follow up measures.

#### 2.5.2. Program Acceptability—Participants

Program acceptability to the participants was assessed through the online process evaluation survey. A total of two questions addressed participant opinions on whether the assessment questionnaires were easy to understand and easy to complete. Six questions addressed delivery and timing of the intervention, with participants asked to rate whether the over the phone consultations were easier, and whether they were more comfortable, than in-person sessions, and whether the number, duration and available booking times of the sessions were appropriate. In total, nine questions addressed participant perceptions of the session content including whether the: information was useful and appropriate; information helped to change behaviour; sessions motivated healthier eating and behaviour change; sessions helped in goal achievement; goals were personalised; suggested coping strategies addressed individual barriers to change; information provided was easy to understand; and whether the session summary emails were useful. Responses were assessed using a five-point Likert scale ranging from ‘strongly agree’ to ‘strongly disagree’ with an additional open-response question included for each section to capture any further feedback. Participant satisfaction was assessed via five questions. A total of four questions asked participants about their perceptions of the intervention providers, including asking participants to rate whether they were knowledgeable, had good communication skills, and whether they felt comfortable to ask questions of the intervention providers on a five-point Likert scale from ‘strongly agree’ to ‘strongly disagree’, with an additional open-response question to provide any further comments on the intervention providers. One final question asked participants to rate their overall satisfaction with the intervention on a five-point Likert scale with responses ranging from ‘extremely satisfied’ to ‘not satisfied at all’.

#### 2.5.3. Program Acceptability—Intervention Providers

Program acceptability to the two intervention providers was assessed through phone interviews. The phone interviews were undertaken by an undergraduate Nutrition and Dietetics student completing an Honours research program (Y.Y.), who was not part of the intervention development or delivery. The semi structured interviews comprised of 12 topic questions and took approximately 15 minutes. The questions were developed by the research team for use in this study. Two questions focused on training, six addressed consultation delivery (i.e., format, timing, engagement and content appropriateness), one question enquired about improvements that could be made for future sessions, two questions asked intervention providers to compare and contrast the addictive eating intervention with other eating behaviour interventions, and the final question asked the intervention providers for any further comments or feedback they had regarding the intervention.

### 2.6. Statistical Analysis

Data manipulation and statistical analyses were completed using Microsoft Excel version 16.62 (Microsoft Cooperation, 2019, Seattle, WA, USA) and Stata statistical software version 14.2. To assess adherence to scheduling, the number of days between baseline (session one) and session two, and between baseline and session three were calculated and compared to scheduled session dates outlined in the protocol. Wilcoxon signed rank tests were used to compare whether the timing of sessions was significantly different than the scheduled timing, between and within groups, with significance set at *p* < 0.05. The median and interquartile range (IQR) of the number of days since baseline are used in reporting as the data was not normally distributed. Data from the participant process evaluation survey are reported as frequency and percentage for quantitative questions, and narratively for open-response questions. Responses to open-ended questions were independently reviewed by two researchers (Y.Y., L.K.C.). Data from the phone interviews with intervention providers are reported narratively. Responses were independently reviewed by two researchers (Y.Y., T.B.) and coded for common themes to identify and categorise key observations for reporting for each question asked.

## 3. Results

### 3.1. Participants

**Figure 1:**
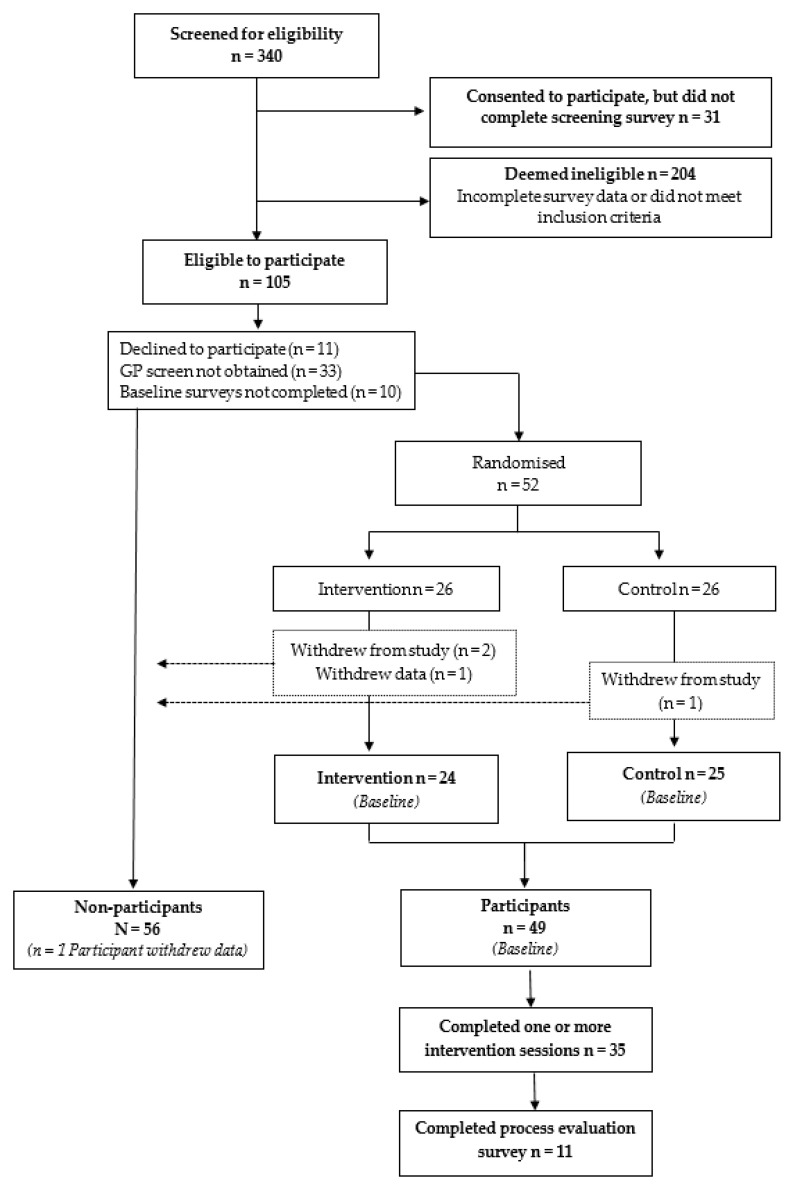
Study flow diagram of participants in the FoodFix intervention randomised controlled trial.

Following eligibility assessment, a total of 52 participants were randomised into either the intervention (n = 26) or wait-list control (n = 26) group (Figure 1). The mean ± SD age of participants at baseline was 43.6 ± 12.2 years, the percentage of female participants was 94%, and most were of moderate socio-demographic background (based on Index of Relative Socio-Economic Disadvantage (IRSD)) [29]. The percentage of participants in each category of addictive eating based on the YFAS 2.0 was 18.4% mild, 6.1% moderate, and 75.5% severe. Participants’ mean ± SD BMI was 36.7 ± 6.8 kg/m^2^. The dominant personality profiles of participants were 22% depression prone, 19% anxiety prone, 8% sensation seeking and 6% impulsive, determined using the Substance Use Risk Profile Scale (SURPS) [26]. There were no significant differences between intervention and control or completers and non-completers based on demographics, addictive eating severity or personality profiles.

### 3.2. Intervention Delivery

Of the intervention group participants, 24 participants (92%) completed session one, 22 (85%) completed session two, and 20 (77%) completed session three. In terms of dropout, two participants in the intervention group did not start the intervention, two were lost to follow-up (i.e., could not be contacted), one discontinued the intervention (not ready for change), and three participants completed all sessions but did not complete follow-up measures. Of the wait-list control group participants who were offered the intervention after the 3-month follow up, 17 participants (65%) completed sessions one and two, and 15 participants (58%) completed session three. Of these, two participants were lost to follow-up (i.e., could not be contacted) and one did not complete follow-up due to technological issues with accessing surveys.

Table 1 presents the number of days since baseline for the scheduled and actual intervention sessions, for sessions two and three. The median days since baseline for session two significantly exceeded the scheduled number of days by two for both intervention and control groups (16 days versus 14 days) (*p* < 0.05). The median days since baseline for session three significantly exceeded the scheduled number of days by nine for the intervention group (51 days versus 42 days) and by 13 for the control group (55 versus 42 days) (*p* < 0.05). There was no statistically significant difference between groups (*p* > 0.05).

In the intervention group, 17 of the 22 participants who completed session two (77%) were defined as adhering (±1 week of scheduled session date) to scheduling protocol and 10 of 20 participants (50%) for session three. For the wait-list control group, 14 of the 17 participants who completed session two (82%) were defined as adhering to scheduling protocol, and five of 15 participants (33%) for session three.

### 3.3. Program Acceptability—Participants

In total, 11 participants completed the process evaluation survey; 21% of the total sample or 31% of those who completed the intervention. Results of the process evaluation survey are presented in Table 2. The majority of participants agreed or strongly agreed that the pre-treatment questionnaires were easy to understand (n = 10, 91%) and easy to complete (n = 8, 73%). In the open-response questions participants stated that the questionnaires were long and repetitive, for example: “*longer than I felt I had the emotional energy to engage with,*” while another participant commented on the need to have more open text space to: “*allow me to give answers that reflected my experience with food addiction.*” For intervention delivery, most participants agreed or strongly agreed that telehealth sessions were easier than in-person sessions (n = 10, 91%) and that the duration of sessions was appropriate (n = 10, 91%), while almost half were neutral that the number of sessions was sufficient (n = 5, 45%). One participant stated: “*I feel like a 4th or 5th consultation would really help me…*” For intervention content, most participants agreed or strongly agreed that the intervention content was useful and helpful (n = 10, 91%) and that the goals were personalised to their needs (n = 10, 91%), with one participant stating: “*I feel like it helped me understand my eating behaviours and gave me genuinely useful methods to overcome barriers! I really enjoyed the proactive approach and setting reasonable, measurable and achievable goals…*” The majority of participants were neutral that the sessions helped them to achieve their goals (n = 7, 64%). In regards to the intervention providers, the majority of participants (n = 10, 91%) agreed or strongly agreed that the intervention providers were knowledgeable and had good communication skills, and that they felt comfortable asking them questions. One participant stated: “*The non-judgmental environment was crucial to being able to effectively explore the barriers to positive food choices.*” For overall satisfaction with the intervention, 64% of participants (n = 7) were extremely or very satisfied, 18% (n = 2) were moderately satisfied, and 18% (n = 2) were slightly satisfied.

### 3.4. Program Acceptability—Intervention Providers

Both intervention providers participated in an interview. Both agreed that they received adequate training which allowed them to implement the intervention sessions as planned, with one stating that the practical face-to-face component of sitting in on sessions was the most helpful element. Both intervention providers expressed that the overall session format, timing, engagement, and content was appropriate for participants. Both reported that the most helpful part of the program was the affirmation of the concept of addictive eating for the participants, as well as using tools to provide feedback that were tailored to participants personality.

With respect to session timings, both intervention providers suggested increasing the allocated time for session one to allow more time for participants to share their addictive eating background, to build rapport, and to answer participants questions. With respect to intervention delivery, one intervention provider preferred the VSee telehealth platform for its ability to convey body language and silences, while the other preferred over-the-phone consultations for its ease of use with participants with poor internet connection.

Both intervention providers stated that the intervention content addressed participant concerns, however they also reported that there was a need to reiterate to participants that FoodFix was not a weight loss program. For future interventions, both intervention providers identified that more sessions would be beneficial. One intervention provider stated that more information around triggers to food consumption would be useful for participants. One intervention provider also expressed that they received positive feedback from participants regarding the coping strategies provided: “*[the] majority of these people they felt more in control… more awareness of what was going on and a couple of extra coping strategies, so they felt like they were more in control of being able to manage it.*”

When asked how addictive eating consultations compared to and contrasted with weight loss and binge eating disorder consultations, both intervention providers described FoodFix as being easier to implement. This was due to the structured nature of FoodFix and the increased motivation participants exhibited for adhering to the intervention. One intervention provider commented on the ease of transferring alcohol and drug addiction interventions to addictive eating, stating that it was, “*interesting to transfer it [the Quik Fix program] into food and to see how comparable it was, that you could use a lot of stuff to do with alcohol and drugs in the same way as food and hearing what people would say, you could just replace the word food with alcohol and the sentence would sound exactly the same.*”

## 4. Discussion

This randomised controlled trial assessed the feasibility of FoodFix, the first personality targeted intervention for the treatment of addictive eating behaviours in Australian adults. Assessment of intervention delivery found that most participants adhered to scheduled timing for session two of the intervention and almost half adhered for session three. Although completion of the process evaluation survey by participants was low (21%), the majority of participants were satisfied with the FoodFix intervention overall and gave positive ratings in terms of content, session delivery and timing, and intervention providers. Assessment of program acceptability to the intervention providers identified that they felt adequately trained to deliver the intervention, and that the overall session format, timing, and content of FoodFix was appropriate for participants. Overall, the feasibility findings for the FoodFix intervention were positive and provide support and direction for the future development and evaluation of the intervention.

In the FoodFix intervention study, the majority of participants adhered to scheduling for intervention session two (77% in intervention and 82% in wait-list control group), however, adherence dropped to 50% and 33%, respectively, for session three. Statistically, the timing of sessions was significantly different than scheduled for intervention and control groups, but was not significantly different between groups. The overall attrition rate for the Foodfix study was 33%. This attrition rate is similar to binge eating disorder treatments delivered through a range of modalities (3–41%), and various cognitive behavioural therapy modalities (including e-therapy) for eating disorders treatment (22–27%) [30,31]. By allowing participants to schedule and reschedule appointments independently on an online scheduling appointment system, the FoodFix study provided increased flexibility to participants and achieved similar retention rates to that of programs requiring more formal scheduling by personnel. Patient self-scheduling may therefore provide a less labour-intensive alternative to traditional scheduling approaches.

Key findings in terms of program acceptability of the FoodFix intervention were similar across participants and intervention providers. Overall, the telehealth model of intervention delivery was acceptable to both participants and intervention providers. Participants identified that the telehealth sessions were more convenient than attending sessions in-person, which may be due to not having to travel to attend the sessions or preferring to have the consultation in a familiar and comfortable space (i.e., their home). Systematic reviews of electronic and mobile health (e&mHealth) interventions for alcohol, drug and problematic gambling addictions have also demonstrated high feasibility and acceptability of e&mHealth delivery; highlighting ease, convenience and the potential for enhanced accessibility as some of the major benefits [32,33]. With approximately 15–20% of the population affected by addictive eating, telehealth’s potential to eliminate geographic barriers to evidence-based care is especially pertinent [9]. Providing alternative communication modalities allows individuals to engage with addictive eating treatment preferentially and may encourage patient adherence [34]. Furthermore, technology-based delivery methods may also help to circumvent specific obstacles that curb help-seeking for addiction, such as the need for anonymity or autonomy, and the stigma that is associated with addictions [33]. There were however, a small proportion of participants who neither agreed nor disagreed that the telehealth delivery was easy or comfortable compared with in-person, suggesting that some may still prefer traditional in-person delivery. Overall, the evidence supports technology-based delivery as a feasible and acceptable means of delivering addictive eating treatment.

Both participants and intervention providers reported positive feedback regarding the personalisation of FoodFix. FoodFix focused on providing advice and feedback to participants based on personality profiles determined from pre-program assessments. This is a unique approach in nutrition that is more commonly found in the treatment of addiction [35,36]. For example, personality-targeted interventions have been found to effectively target modifiable risk factors associated with higher risk of initiation and development of substance use disorders [35,36]. Another key element of the FoodFix intervention was collaborative goal setting. Process evaluation findings highlight that the participants felt their goals were personalised and therefore rated this element highly. Goal setting as a behaviour change technique has demonstrated efficacy in interventions for various other health conditions, for example mental health conditions [37] as well as weight management [38]. However, in the current study most participants also felt that the intervention sessions themselves did not assist them to achieve their goals. This is potentially a limitation of the number of intervention sessions and the amount of content covered within each, in that additional sessions would allow more time for participants and providers to review set goals and work towards goal achievement. The inclusion of additional intervention sessions was suggested by both participants and intervention providers, while intervention providers also suggested a longer initial session. The feedback indicated that this may be beneficial to allow participants adequate time to share their individual experience with addictive eating, for addressing entrenched addictive eating behaviours and further supporting participants mental health. Overall the key elements and design of the FoodFix intervention were deemed acceptable to participants and intervention providers.

A key finding from the process evaluation was that FoodFix participants found the pre-program questionnaires to be long, repetitive, and in some cases did not adequately capture their addictive eating status or behaviours. Addictive eating is an emerging field with ongoing debate around constructs, classification, and treatment [6]. Therefore, existing questionnaires are likely unable to capture all aspects of addictive eating, particularly as the experience differs from one individual to another. In recent years there has been the emergence of alternative tools in addition to the YFAS, such as the addiction-like eating behaviour scale which attempts to quantify addiction-like eating behaviour [39]. However, the addiction-like eating behaviour scale captures addictive eating as an ‘eating addiction’ rather than a form of substance dependence [39]. Whilst addictive eating involves both behavioural (i.e., eating) and substance-related (i.e., food) symptoms, a systematic review by Gordon et al. has implicated that addictive eating closely follows the pattern of substance-use disorders [6,40]. The success of FoodFix in helping participants to address their addictive eating behaviours indicates that one plausible mechanism for the treatment of addictive eating follows along the same modality as those for alcohol and drug addiction interventions. The current study also highlights the need for more tools to adequately assess and capture all aspects of living with addictive eating. This would allow participants to elaborate on their experiences with addictive eating and improve clinician understanding of each participant’s needs and challenges in order for them to provide personalised treatment. Relative to the finding that the pre-program questionnaires were burdensome for participants; this could also be a contributor to the low completion rates for the process evaluation survey as this was in addition to other data collection surveys. Ensuring only relevant and key data are collected, and that data collection procedures are streamlined and make use of any objective and automated measures and processes as much as possible may help to address this in future research and practice.

The strengths of this study include the relatively high participant retention rate in terms of assessing participant adherence to scheduled intervention sessions, and the assessment of program acceptability from both the participant and intervention provider perspectives. The limitations of this study include the small sample size, including for the process evaluation data and there being only two intervention providers. This small sample size and the voluntary nature of the process evaluation survey limited the ability to analyse the collected data, including comparing adherence and acceptability across key characteristics such as food addiction severity and personality types. The qualitative results obtained within the process evaluation survey were also limited in number for some components, including the intervention content. The low completion rate of the process evaluation survey could be in part due to the mental health comorbidities experienced by most participants, which influences their motivation for activities of daily living and could also have influenced study participation. Further, the participant sample were predominantly female, middle-aged, and of moderate socio-economic background, which may limit the generalisability of the findings. Most measures in this study were self-reported and therefore there is the potential for self-reporting bias. In addition, consultation durations could not be obtained from the telehealth platform. Therefore, actual durations could not be assessed and compared to planned durations. In terms of recommendations for future research, future addictive eating intervention studies should aim to include larger sample sizes that include more representation of both sexes. Future studies should also include a detailed process evaluation incorporating a mixture of objective and qualitative measures where possible, to inform the continued development of evidence-based treatments for addictive eating that align with both successful behaviour change theories and patient experience. The usefulness and insight from the qualitative findings in the current study also highlight the importance of qualitative research methods for capturing all aspects of addictive eating, and support the need for more qualitative research in the area of addictive eating [41,42,43].

## 5. Conclusions

This study reports the feasibility findings of the FoodFix intervention, the first personality targeted intervention for the treatment of addictive eating behaviours. Overall the key elements and design of the FoodFix intervention were deemed acceptable to participants and intervention providers. Elements for further development and evaluation were also identified, including the ideal number of intervention sessions. The findings of this study add to the literature demonstrating the feasibility of adapting and translating substance-related addictive disorder treatment methods to addictive eating.

## Figures and Tables

**Table 1:** Study records of timing (days since baseline) of intervention sessions in the FoodFix intervention randomised controlled trial.

Intervention Session		
Session Two	Intervention (n = 22)	Control (n = 17)
Scheduled days since baseline	14	14
Median (IQR) days since baseline	16 (14–18) ^a^	16 (14–21) ^a^
Number (%) of participants with good adherence to session scheduling	17 (77)	14 (82)
Number (%) of participants rescheduling ^b^	2 (9)	2 (12)
Session Three	Intervention (n = 20)	Control (n = 15)
Scheduled days since baseline	42	42
Median (IQR) days since baseline	51 (44–68) ^a^	55 (48–69) ^a^
Number (%) of participants with good adherence to session scheduling	10 (50)	5 (33)
Number (%) of participants rescheduling ^b^	2 (10)	4 (27)

^a^ Indicates median days since baseline was significantly different than scheduled (*p* < 0.05). ^b^ Refers to total number of participants who rescheduled and does not reflect the number of times a participant may have rescheduled.

**Table 2:** Process evaluation questionnaire responses from participants in the FoodFix intervention randomised controlled trial (n = 11).

	n (%)				
Process Evaluation Items	Strongly Agree	Agree	Neutral	Disagree	Strongly Disagree
**Pre-treatment questionnaire**					
The questionnaires easy to understand	4 (36)	6 (55)	1 (9)	0 (0)	0 (0)
The questionnaires easy to complete	4 (36)	4 (36)	2 (18)	1 (9)	0 (0)
**Intervention delivery and timing**					
Over the phone was easier than in person	7 (64)	3 (27)	1 (9)	0 (0)	0 (0)
The number of sessions was sufficient	1 (9)	4 (36)	5 (45)	1 (9)	0 (0)
The session durations were appropriate	4 (36)	6 (55)	1 (9)	0 (0)	0 (0)
The availability of times for sessions was suitable (n = 9)	5 (56)	2 (22)	2 (22)	0 (0)	0 (0)
Compared with in person, I felt comfortable interacting over the phone	4 (36)	5 (45)	2 (18)	0 (0)	0 (0)
**Intervention content**					
The information provided was useful and helpful	4 (36)	6 (55)	1 (9)	0 (0)	0 (0)
The information provided helped me change my behaviours	2 (18)	5 (45)	3 (27)	1 (9)	0 (0)
The sessions motivated me to eat better and make changes	3 (27)	5 (45)	2 (18)	1 (9)	0 (0)
The sessions helped me achieve my goals	2 (18)	1 (9)	7 (64)	1 (9)	0 (0)
The goals were personalised to my needs	4 (36)	6 (55)	1 (9)	0 (0)	0 (0)
The strategies suggested by the dietitian addressed the barriers preventing me from changing my eating behaviours	3 (27)	4 (36)	3 (27)	1 (9)	0 (0)
The information provided was easy to understand	7 (64)	4 (36)	0 (0)	0 (0)	0 (0)
I found the summaries I received after the sessions useful	4 (36)	6 (55)	1 (9)	0 (0)	0 (0)
**Intervention providers**					
The dietitian was very knowledgeable	1 (9)	8 (73)	1 (9)	0 (0)	1 (9)
The dietitian had good communication skills	1 (9)	8 (73)	1 (9)	0 (0)	1 (9)
I felt comfortable asking the dietitian questions	1 (9)	8 (73)	1 (9)	0 (0)	1 (9)

## Supplementary Materials

The following are available online at https://www.mdpi.com/2076-328X/10/12/186/s1, Table S1: Description of the FoodFix intervention sessions.
