# Supplementary material for: Process Evaluation of a Personality Targeted Intervention for Addictive Eating in Australian Adults
Source: Behav Sci (Basel). 2020 Dec 3;10(12):186. doi: 10.3390/bs10120186 (PMC7761794; doi:10.3390/bs10120186)
Supplement: Supplementary file 1 [file behavsci-10-00186-s001.pdf]

**Table S1** Description of the FoodFix intervention sessions

| Session | Content |
|---|---|
| **Session 1 (45 min)** | • Feedback on baseline scores of addictive eating (YFAS 2.0) and dietary intake (AES)<br>• Identify 3 goals for dietary change using motivational interviewing. Goals regarding dietary behaviour were personalised around dietary feedback from the Australian Eating Survey and evaluated in comparison to national dietary recommendations. Goals could be related to both core and non-core foods / beverages.<br>• Determine a 'food line' to identify when eating was no longer enjoyable or no longer tasting foods consumed. Prompts: frequency, quantity, timing, amount of food<br>• Identify what constitutes a 'good eating occasion' and discuss strategies to stay below the "food line" using a range of 13 strategies or strategies suggested by the participant<br>• Enablers and barriers to change<br>• Discuss harm minimisation strategies to assist in development of goals<br>• Assess confidence to achieve goals (1-10 scale) |
| **Session 2 (25-30min)** | • Review session 1, discuss enablers and barriers<br>• Check in for periods of overeating, assess scale for change<br>• Feedback on major personality style (SURPS) from baseline surveys including anxiety-prone, depression-prone, impulsivity, sensation seeking<br>• Coping skills training based on personality<br>• Integrate skills training with good eating occasion<br>• Assess confidence and practice skills training in mind to increase confidence |
| **Session 3 (15-20 min)** | • Check in/ brief problem solve and encourage to continue with goals and strategies |

YFAS; Yale Food Addiction Scale, AES; Australian Eating survey, SURPS; Substance Use Risk Profile Scale.
